# Exploring *Psilocybe cubensis* Strains: Cultivation Techniques, Psychoactive Compounds, Genetics and Research Gaps

**DOI:** 10.3390/jof11020099

**Published:** 2025-01-28

**Authors:** Eyal Kurzbaum, Tomáš Páleníček, Amiel Sharchaton, Sara Azerrad, Yaron Dekel

**Affiliations:** 1 Water Science Department, Tel-Hai College, Upper Galilee 1220800, Israel; 2 Shamir Research Institute, University of Haifa, P.O. Box 97, Qatzrin 1290000, Israel; eyal2@gri.org.il (A.S.); saraa@gri.org.il (S.A.); yarond@gri.org.il (Y.D.); 3 Department of Geography and Environmental Studies, University of Haifa, Mount Carmel, Haifa 3498838, Israel; 4 National Institute of Mental Health, 250 67 Klecany, Czech Republic; tomas.palenicek@nudz.cz; 5 3rd Faculty of Medicine, Charles University, 100 00 Prague, Czech Republic; 6 The Natural Resources and Environmental Research Center-NRERC, University of Haifa, Mount Carmel, Haifa 3498838, Israel; 7 The Cheryl Spencer Department of Nursing, The Cheryl Spencer Institute of Nursing Research, University of Haifa, Haifa 3498838, Israel; 8 The Cheryl Spencer Institute of Nursing Research, University of Haifa, Haifa 3498838, Israel

**Keywords:** *Psilocybe*, psychoactive, psilocybin, psilocin, fungi, mushroom, mycelium, genetics

## Abstract

*Psilocybe cubensis*, a widely recognized psychoactive mushroom species, has played a significant role in both historical and modern therapeutic practices. This review explores the complex interplay between genetic diversity, strain variability and environmental factors that shape the biosynthesis of key psychoactive compounds, including psilocybin and psilocin. With many strains exhibiting substantial variability in their phenotypic characteristics and biochemical content, understanding and documenting this diversity is crucial for optimizing therapeutic applications. The review also highlights advances in cultivation techniques, such as submerged fermentation of the mycelium, and innovative analytical methodologies that have improved the precision of compound quantification and extraction. Although there is limited scientific information on *P. cubensis* due to nearly four decades of regulatory restrictions on psychedelic research, recent developments in genetic and biochemical studies are beginning to provide valuable insights into its therapeutic potential. Furthermore, this review emphasizes key knowledge gaps and offers insights into future research directions to advance the cultivation, scientific documentation of strain diversity, regulatory considerations and therapeutic use of *P. cubensis*.

## 1. Scientific Background and Historical Significance

The study of psilocybin-producing fungi has gained significant momentum over the past decade, reflecting a surge in scientific interest and research efforts. According to publication data retrieved from PubMed [1], the keyword ‘psilocybin’ has seen a sharp increase in publications in recent years, particularly from 2020 onward, with a peak in 2024 (480 publications) and a strong continuation into 2025 (39 publications so far). This surge likely reflects renewed scientific and public interest in psilocybin’s potential therapeutic applications, particularly in mental health treatments.

Between 1960 and the early 1970s, research activity was moderate, with a notable peak in 1968 (39 publications), reflecting early psychedelic research before legal restrictions were imposed. A subsequent decline in publications is evident from the 1970s through the 1990s, coinciding with stricter regulations and diminished academic interest. The 1980s and 1990s show relatively low but steady research output, often fewer than 10 publications per year. From the early 2000s, a gradual resurgence is visible, with publication numbers steadily rising, particularly post-2015, aligning with the growing acceptance of psychedelic-assisted therapy. The exponential growth from 2019 onward suggests that psilocybin research is currently at an all-time high, likely driven by policy shifts, increased funding and promising clinical trial results. Overall, this trend highlights a remarkable revival in psilocybin research, transitioning from historical suppression to a modern scientific renaissance.

The renewed scientific focus on these fungi is particularly evident in studies on *P. cubensis*, which explore its domestication, genetic diversity and biochemical properties.

Hallucinogenic mushrooms such as *Psilocybe* spp. have been revered by indigenous cultures across the globe for millennia, utilized in sacred rituals to induce profound spiritual and healing experiences. The *Psilocybe* genus is well known for the synthesis of psychoactive compounds such as psilocybin, psilocin, baeocystin and aeruginascin [2], and *P. cubensis* is one of the most widely recognized and cultivated psilocybin mushrooms in the world. Although it is distributed across different continents, this species was first documented in 1906 in Cuba, from which it derives its name. According to Singer and Smith [3], *P. cubensis* is characterized by the following morphological features: a cap (pileus) 16–80 mm wide that evolves from conic–campanulate to convex and flat, with a color change from white to ochraceous-brown. The gills (lamellae) are pale gray, later deepening to sepia, and are adnate to adnexed. The stem (stipe) measures 20–80 mm in height and is hollow and yellowish, darkening centrally. The spores are elliptic, 8.8–10.5 microns wide, with a germ pore and yellow-brown tint in KOH solution. These characteristics facilitate accurate identification and understanding of the species’ developmental stages.

*P. cubensis* is a coprophilous species, meaning it thrives on the dung of herbivorous animals such as cows and horses. It prefers humid environments, making subtropical regions, particularly river valleys, ideal for its growth. Due to its relatively high psilocybin content and widespread natural occurrence, *P. cubensis* has garnered more attention than other hallucinogenic mushrooms among both scientists and growers. In addition, this species is preferred due to its ease of cultivation in laboratory conditions and at home, its ability to thrive on readily available substrates and its tendency to produce large fruiting bodies [4].

Modern research suggests that psychoactive compounds, such as psilocin, have high potential to catalyze therapeutic transformations in treating resistant psychiatric disorders such as depression, anxiety and post-traumatic stress disorder (PTSD) and addiction [5,6,7,8,9]. Due to positive indicators for treating these psychiatric conditions, interest in psilocybin mushrooms has increased in recent years, with *P. cubensis* receiving particular attention as it is relatively easy to cultivate. However, despite this growing interest, there is limited scientific research on the effects of growth conditions on the psychoactive compound content. Although information on cultivating different strains exists within the community of amateur mycologists, there are few scientific studies in the professional literature. This review focuses exclusively on research conducted and published according to scientific standards.

With dozens of strains of *P. cubensis*, each exhibiting notable differences in psilocybin and psilocin content and growth characteristics, understanding and documenting these variations is essential. This review aims to provide a comprehensive, up-to-date summary of the cultivation techniques and growth conditions used and their effect on the psychoactive compound content of the fungus and highlight the key research gaps related to *P. cubensis*.

## 2. Strain Variability

**Figure 1:**
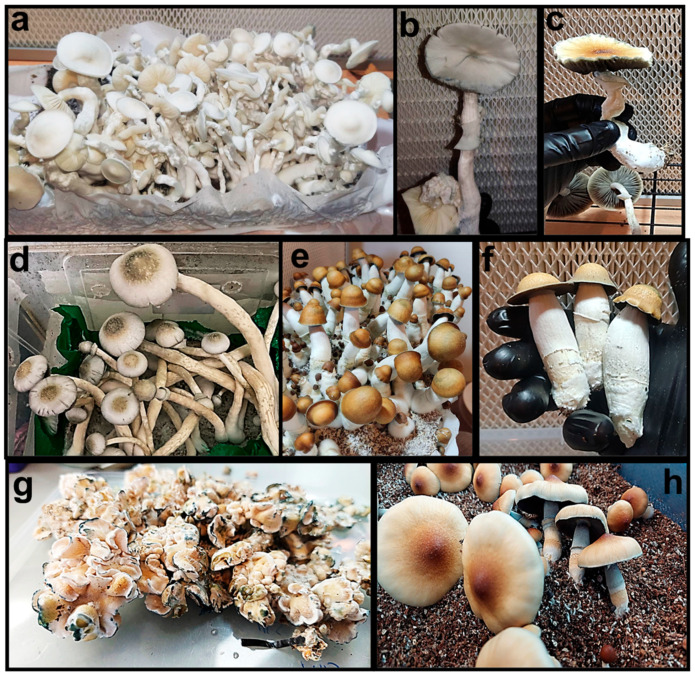
Mature fruiting bodies of different *P. cubensis* strains. All strains were grown on a solid organic medium under controlled conditions. (**a**) Monotub of ‘Psilocybe fanaticus albino’ (P.F.A); (**b**) one fruiting body of ‘Psilocybe fanaticus albino’ (P.F.A); (**c**) ‘Cambodian’; (**d**) ‘Long Albino Penis Envy’; (**e**) Monotub of ‘Penis Envy 7’; (**f**) Fruiting body of ‘Penis Envy 7’; (**g**) ‘Enigma’; (**h**) ‘Z’ (© photos by the authors).

*P. cubensis* is characterized by significant diversity in phenotypic strains, each distinctly developed through systematic mycological research and robust engagement by psychedelic mushroom enthusiasts (Figure 1). This proliferation is primarily driven by extensive cultivation experiments and selective breeding within these communities. Despite the informal recognition of dozens of these strains among practitioners, there remains a compelling imperative for formal academic documentation and rigorous scientific investigation to fully classify and comprehend these variants [10,11].

Prior to detailing specific strains, it is important to acknowledge that the following examples are predominantly sourced from non-academic, community-based reports. This is due, in part, to legislative restrictions on psilocybin, which have limited formal academic research on *Psilocybe* species. The following descriptions provide a nuanced understanding of the variety within *P. cubensis* strains. Among the most prominent strains, ‘Golden Teacher’ is renowned for its vigorous growth and substantial psychotropic effects, garnering substantial preference among both novices and experienced mycologists. The ‘B+’ strain is lauded for its resilience and consistent productivity under diverse environmental conditions. Furthermore, the ‘Penis Envy’ strain is noted for its exceptional potency and distinct morphological characteristics, which have contributed to its recognition and prominence within the community.

Additional common strains include the ‘Albino A+’, noted for its distinctive white, leucistic fruiting bodies; the ‘Mazatapec’, which is connected to the rich spiritual traditions of the Mazatec region in Mexico; the ‘Cambodian’, known for its swift colonization and prolific fruiting; and the ‘Amazonian’, sought after for its large, potent mushrooms. The ‘Enigma’ strain attracts attention due to its unique, blob-like formations that deviate from typical mushroom shapes, while the ‘Jack Frost’ is recognized for its rapid growth and high potency. Other known strains with relatively high psilocybin content include the ‘Texas Yellow’, the ‘Thai Cubensis’, the ‘B+’, the ‘Blue Meanie’, the ‘Creeper’ and more [12].

There is notable intra-strain and inter-strain variability in the growth rates and concentrations of psychoactive compounds, such as psilocybin and psilocin. Intra-strain differences refer to the variability found within a single strain, where individual mushrooms may show different psilocybin levels or growth characteristics due to environmental factors or minor genetic mutations. Inter-strain differences, on the other hand, are observed between different strains of the same species, reflecting broader genetic variations that influence the overall potency, growth rates and environmental tolerances among strains [12]. This variability, influenced by both genetic and cultivation factors, critically affects the efficacy and safety of psilocybin-based treatments in clinical applications [11]. As a result, comprehensive genetic and environmental studies are essential to optimize the production of these compounds for medical purposes, ensuring consistency and reliability in therapeutic contexts. Advanced genetic mapping techniques have begun to elucidate the specific genes involved in the biosynthetic pathways of these compounds, opening avenues for the genetic optimization of strains. Such enhancements aim to increase yields and tailor specific therapeutic properties, essential for breeding programs dedicated to developing strains with defined psychoactive profiles for varied therapeutic contexts. The integration of traditional cultivation methods with these advanced genetic insights is poised to significantly advance the field of mycology, shedding light on the intricate relationships between genotype, phenotype and environmental factors in *P. cubensis* [13,14,15].

Table 1 provides a comprehensive overview of the quantification of psilocybin and psilocin in various strains of *P. cubensis*, highlighting differences in compound concentrations across diverse growth conditions and sources. It includes strains sourced from both laboratory-grown and wild specimens, with detailed descriptions of their origins, cultivation methods and measured alkaloid concentrations. Psilocybin levels range from trace amounts to 19.9 mg/g, while psilocin concentrations vary widely, with some samples showing no detectable levels. The data span multiple decades and geographic locations, illustrating the influence of environmental and controlled conditions on compound production. In addition, it seems that laboratory-grown specimens generally display more consistent concentrations compared to wild strains, which exhibit higher variability, likely due to environmental influences.

Although Table 1 focuses exclusively on *P. cubensis*, we note that Gotvaldová et al. [14] conducted a study focused on other *Psilocybe* species and more, analyzing the biochemical diversity of 226 fruiting bodies collected from diverse geographical regions. The study also included specimens from non-*Psilocybe* genera such as *Gymnopilus*, *Inocybe*, *Panaeolina*, *Panaeolus*, *Pholiotina* and *Pluteus*, although the discussion herein will focus primarily on *P. cubensis*. Specifically, this subset of *P. cubensis* was investigated for its range of key psychoactive alkaloids, demonstrating considerable variability among different strains of *P. cubensis*. The concentrations of psilocybin varied from 0.208 to 5.344 mg/g, psilocin from 0.651 to 3.509 mg/g, baeocystin from 0.139 to 0.881 mg/g, norbaeocystin from 0.044 to 0.161 mg/g and aeruginascin from 0.026 to 0.053 mg/g. These quantitative findings highlight the significant impact of strain selection (and other parameters such as storage and age of the fruiting bodies) on the therapeutic efficacy and safety of psilocybin in clinical settings, underscoring the necessity for detailed characterizations of psychoactive profiles in ongoing psilocybin research.

Furthermore, the investigation revealed that other *Psilocybe* species may exhibit considerably higher alkaloid concentrations. Notably, *P. serbica var. bohemica* demonstrated exceptionally high levels of psilocybin and psilocin, with concentrations of up to 15.543 mg/g of psilocin, while *P. azurescens* presented up to 17.8 mg/g. However, the cultivation of these species under laboratory conditions poses significant challenges when compared to *P. cubensis*, which may limit their practical application in research and therapeutic contexts. This detailed examination not only deepens our understanding of *P. cubensis* but also expands our knowledge base regarding the wider spectrum of psychoactive fungi, which is imperative for the advancement of therapeutic applications and safety protocols in clinical environments.

## 3. Genetic Insights and Evolutionary Pathways in Psilocybe: Challenges, Potential and Pharmacological Implications

Genomic research on *Psilocybe* is restricted, with genetic information only available for approximately ten strains across primary databases such as MycoCosm [24] and GenBank [25]. This scarcity reflects broader legislative challenges that restrict formal scientific studies of *Psilocybe* species. A comprehensive genetic examination could help clarify various phenomena associated with psilocybin-producing fungi. These include the entourage effect, which suggests that other compounds might synergize with psilocybin to influence psychotropic outcomes, as well as conditions such as wood lover’s paralysis (WLP), an unexplained peripheral paralysis linked to certain lignicolous *Psilocybe* species. While some genomes—such as those of *P. mexicana* and *P. azurescens*—have been sequenced, fragmented data, as well as potential gene cluster gaps, present challenges in correlating genetic profiles with secondary metabolite diversity. Early results indicate a complex genetic landscape with several types of natural product genes, including non-ribosomal peptide synthetases and terpene synthases. Additional in-depth studies are essential to explore these biosynthetic pathways and their potential pharmacological roles [14].

A very recent publication by Bradshaw et al. [26] investigated the evolutionary origins and genetic makeup of psilocybin. The study explored the genetic and biosynthetic pathways in *Psilocybe* and a few other fungi through metagenomic analysis of fungarium specimens and modern genomic sequencing. The psilocybin biosynthetic gene cluster (BGC) was highly conserved in *Psilocybe* species, but variations existed in gene order among two primary *Psilocybe* clades, suggesting evolutionary divergence. Evidence indicated that the psilocybin BGC was likely acquired by horizontal gene transfer (HGT) in other genera, such as *Panaeolus* and *Gymnopilus*, highlighting a complex evolutionary history. Phylogenetic analysis identified two main *Psilocybe* clades, each linked to specific ecological niches, such as wood decay or soil-based saprotrophic lifestyles. Through divergence dating, the origin of the psilocybin BGC was estimated at around 67 million years ago, with subsequent independent acquisitions in other fungi over millions of years [26]. Despite advances, strain-level identification remains limited due to the small number of species analyzed at a genomic level.

**Figure 2:**
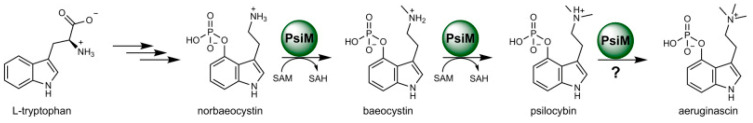
The biosynthetic pathway of psilocybin originates from L-tryptophan, which undergoes decarboxylation, oxygenation and phosphate ester formation to yield norbaeocystin, followed by methylation by the PsiM enzyme. SAM (S-adenosyl-L-methionine) acts as a methyl donor, while SAH (S-adenosyl-L-homocysteine) is the byproduct. A third methyl transfer by PsiM to yield aeruginascin has been proposed. Reprinted from Hudspeth et al. 2024 [33], under a Creative Commons Attribution 4.0 International License.

The identification of the psilocybin BGC has been particularly significant in understanding the genetic factors influencing psilocybin synthesis. Psilocybin is produced through a series of enzymatic reactions facilitated by four genes that are located in close proximity to one another. Collectively known as the Psi genes, they span an approximate genomic region of 11–22 kilobases. Within this region, there are four genes that encode the biosynthetic enzymes: PsiD, PsiK, PsiM and PsiH [27]. The PsiK enzyme phosphorylates the L-tryptophan-derived intermediate, 4-hydroxytryptamine (4-HT), to produce norbaeocystin and can also restore psilocybin from psilocin, both in an ATP-dependent reaction. The crystal structure of PsiK was very recently resolved, and its crystallographic model provides a preliminary basis for future protein engineering [28]. Furthermore, there is one gene, designated PsiT, which is thought to code for a solute transporter, although its exact function remains unclear (see also [13,29,30,31,32]). For a detailed description of the biosynthetic pathway of psilocybin, readers are encouraged to refer to the study conducted by Hudspeth et al. (2024) [33]. The general biosynthetic pathway is shown in Figure 2.

The significance of the psilocybin biosynthetic pathway was recently highlighted in a study suggesting that psilocybin from natural extracts exerts stronger and more prolonged effects on neuroplasticity and metabolic changes compared to synthetic psilocybin. These findings support the hypothesis that additional compounds present in the mushroom extract may enhance or modulate the effects of psilocybin, resulting in a more comprehensive therapeutic profile. Further research is needed to confirm these observations and identify the specific molecules responsible for the enhanced effects of natural extracts [34]. A genomic analysis, conducted to track genetic changes in commercially labeled cultivars, revealed that these often lack genetic similarity across different suppliers [35]. Additional studies indicate that *P. cubensis* cultivars display low heterozygosity and high relatedness, which implies a history of inbreeding. However, mating compatibility in *P. cubensis* may follow a tetrapolar system seen in related fungi, with markers such as mating-type genes that track genetic relationships and compatibility.

This body of research underscores the potential for genetic studies to enhance psilocybin yields across various *Psilocybe* species, providing a valuable resource for optimizing therapeutic applications.

## 4. Cultivation Techniques and Environmental Influences on Psilocybin Production: Present Knowledge and Research Gaps

There is, in fact, very limited knowledge regarding cultivation techniques and environmental influences on psilocybin production in hallucinogenic fungi in general and in *P. cubensis* in particular. It is surprising that the first published study in the academic literature on the biosynthesis of psilocybin and psilocin in *P. cubensis* focused on submerged growth in bioreactors using a liquid medium for mycelial cultivation, rather than on fruiting bodies grown on solid media. Catalfomo and Tyler [21] provided early experimental evidence demonstrating that variations in these conditions during submerged fermentation significantly influenced the yield and quantity of psilocybin. Their findings, which highlighted the importance of controlled pH and nutrient availability, underscored the need for precise environmental regulation to optimize psilocybin production. Their study also emphasized the necessity of developing tailored cultivation protocols to effectively control both growth rates and the accumulation of psychoactive compounds.

Another study by Neal et al. [36] explored the impact of phosphorus levels in growth media and nitrogen metabolism on the accumulation of secondary metabolites in three species: *P. cubensis*, *P. cyanescens* and *Panaeolus campanulatus*. The experiments were conducted in submerged cultures, aiming to determine how varying phosphorus levels affect nitrogen metabolism and the production of active tryptamine derivatives such as psilocybin and psilocin. It was hypothesized that limited phosphorus availability might trigger metabolic pathways leading to secondary metabolite production, similar to the alkaloid synthesis observed in other fungi and plants. Among the three species, *P. cubensis* exhibited the highest capacity to accumulate psilocybin and psilocin, with concentrations reaching up to 2 µg/mg under specific conditions. In contrast, *P. cyanescens* did not accumulate detectable levels of these compounds and showed limited metabolic response to changes in phosphorus availability. *Panaeolus campanulatus* maintained a stable nitrogen pool and secreted nitrogenous metabolites, but failed to produce measurable levels of active tryptophan derivatives such as psilocybin or psilocin. The results suggest that psilocybin production in *P. cubensis* primarily occurs during the stationary growth phase, when nitrogen is no longer required for protein synthesis. Additionally, excess phosphorus may inhibit secondary metabolite production, delaying or suppressing the synthesis of tryptamine derivatives. These findings highlight the importance of balancing phosphorus and nitrogen levels in growth media to maximize the accumulation of active compounds in *P. cubensis*.

Although submerged growth in liquid media was the focus of the earliest studies, more recent research has shifted towards the cultivation of *P. cubensis* on solid media, studying the fruiting bodies rather than the mycelium. However, only a limited number of academic studies have focused on identifying the optimal environmental conditions—such as substrate composition, temperature and humidity—for growing different strains of *P. cubensis*. One of the earliest studies on solid-medium cultivation, conducted by Badham et al. [37], revealed that *P. cubensis* growth is highly influenced by environmental humidity. The study found that maintaining high relative humidity, particularly above 90%, promotes optimal growth while reducing water loss from the fruiting bodies. Conversely, when the vapor pressure deficit exceeded 475 pascals, growth rates decreased significantly, with some mushrooms showing reduced development and lower water content. The study also demonstrated that misting the mushrooms improved hydration, leading to faster growth and increased water transpiration, underscoring the importance of water availability for successful cultivation. Interestingly, exposure to light had no significant effect on growth when compared to mushrooms grown in darkness. Morphological traits also played a role, as mushrooms with thinner stripes or higher surface area-to-volume ratios experienced greater water loss and slower growth rates compared to those with thicker stipes or lower ratios. These findings emphasize the need for careful control of humidity levels and hydration practices throughout the cultivation process to achieve optimal yields.

A study by Gartz and Moller [16] showed that *Psilocybe* spp. mycelium generally contains lower concentrations of psilocybin and psilocin compared to the fruiting bodies. In their study, the fruiting bodies of *P. bohemica* exhibited a range of psilocybin concentrations, from 0.11% to 1.34% by dry weight, with the highest concentrations found in the caps. Interestingly, while psilocybin was detected in cultured mycelium, no other alkaloids were present, suggesting that the biosynthetic capacity of mycelium differs from that of fruiting bodies. Recent research [10,38] further supports these findings, showing that mycelium, although containing lower psilocybin levels, produces a unique array of secondary metabolites absent in fruiting bodies. This underscores the need for targeted, stage-specific cultivation studies to optimize conditions for both mycelial and fruiting body development. Achieving consistent yields of psychoactive compounds such as psilocybin through careful control of growth conditions is crucial, particularly for therapeutic and pharmaceutical applications. Further exploration of the metabolomic profiles of *Psilocybe* spp. revealed notable differences in secondary metabolite production among the growth stages and substrates used [38]; while the mycelium was found to generally contain lower levels of primary psychoactive compounds, it still produced a variety of secondary metabolites with potential psychoactive effects. These include unique alkaloids absent from the fruiting bodies, such as higher concentrations of alpha-glycerylphosphorylcholine, N-acetylglucosamine and trimethylglycine, indicating that the mycelium possesses distinct biosynthetic capabilities. Such findings underscore the importance of considering both mycelial and fruiting body stages in research, as each contributes differently to the overall psychoactive potential of the fungi.

These differences in compound profiles between mycelium and fruiting bodies also have significant implications for pharmacological research. The unique alkaloid profile of mycelium, coupled with its lower psychedelic potential, opens new avenues for developing therapeutic applications of *Psilocybe* spp. that may avoid some of the challenges associated with highly psychoactive compounds. Given this, there is a clear need for further studies to explore the full pharmacological potential of mycelial metabolites.

Submerged fermentation in bioreactors is emerging as a promising method for the large-scale production of mycelium-based products. This technique has already shown considerable potential for medicinal fungi and edible fungi in the alternative protein industry. Submerged fermentation allows for precise control of overgrowth conditions, such as oxygen levels and nutrient availability, enabling the production of consistently safe fungal biomass. The scalability and reproducibility of this approach make it highly attractive for both food and biotechnology applications, including the production of mycelium-based meat alternatives.

For *Psilocybe* species, submerged fermentation holds the potential for producing mycelium-based psilocybin products more efficiently than traditional solid-medium methods. However, research on the submerged cultivation of *P. cubensis* remains limited. Given the potential for more consistent, scalable psilocybin production in bioreactors, further investigation is urgently needed to optimize this method for pharmaceutical use. Submerged fermentation could prove particularly valuable for industrial-scale production, given its ability to meet regulatory standards and ensure consistency across batches.

A review of the key differences in gene regulation and metabolite profiles between submerged fermentation and fruiting body production in edible fungi noted that mycelium in submerged cultures experiences distinct environmental conditions—such as oxygen levels, humidity and shear stress—compared to that in solid media [39]. These conditions affect gene activation, which in turn influences growth and metabolite production. While fruiting bodies form under specific environmental triggers such as light and temperature shifts, which activate reproductive genes, mycelium in submerged cultures remains in a vegetative state. This distinction in gene regulation results in differing metabolite profiles between the two growth stages. Although these phenomena have been well studied in non-hallucinogenic fungi, similar research on hallucinogenic fungi such as *Psilocybe* is still in its early stages [40]. Expanding this research could lead to breakthroughs in producing consistent, safe psilocybin products using submerged fermentation techniques.

While solid-medium cultivation of *P. cubensis* can effectively produce fruiting bodies, it is a labor-intensive process that requires careful control of environmental factors such as humidity, temperature and light. Furthermore, variability in substrate composition and environmental fluctuations can lead to inconsistencies in metabolite concentrations. While solid-medium cultivation is suitable for small-scale production, it is less practical for industrial applications where standardization and scalability are essential [41].

**Figure 3:**
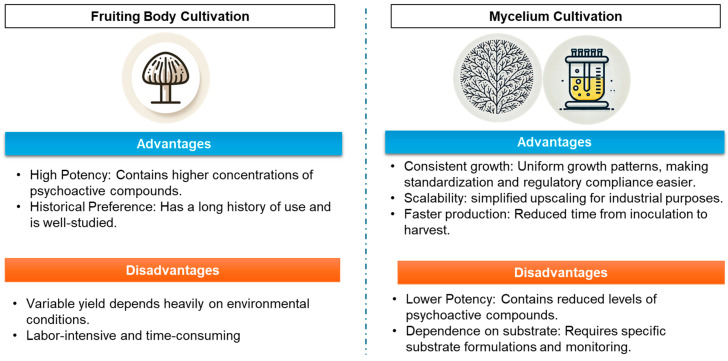
Comparison of two cultivation methods for *Psilocybe* spp., summarizing their respective advantages and disadvantages.

**Figure 4:**
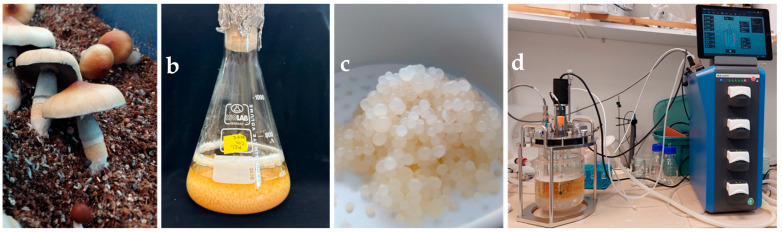
*Psilocybe cubensis* ‘Z’ grown under different conditions: (**a**) The typical morphology grown on coconut coir; (**b**) submerged cultivation in shaken flasks; (**c**) mycelial pellets of mycelium grown for 7 days in a rotary shaker; (**d**) submerged cultivation in a lab-scale fermenter (© photos by the authors).

In conclusion, both mycelial and fruiting body cultivation offer distinct advantages and challenges for psilocybin production. Mycelium, particularly when grown using submerged fermentation, holds promise for scalable, consistent production, while fruiting bodies remain a reliable source of high psilocybin content. Figure 3 and Figure 4 summarize a comparison of the two cultivation methods for *Psilocybe* species, highlighting their respective advantages and disadvantages. Further research into both growth methods is essential to fully understand the biosynthetic potential of *Psilocybe* spp. and develop optimized cultivation protocols for therapeutic and industrial applications.

## 5. Optimized Extraction Methods and Analytical Techniques

The development of optimized extraction methods has significantly improved the efficiency and reliability of psilocybin extraction from *P. cubensis*. In a study by Gotvaldová et al. [42], methanol combined with acetic acid (0.5% *v*/*v*) emerged as the most effective choice for extraction. The study found that homogenizing dried mushroom powder significantly improved the extraction yield of tryptamines compared to using whole pieces. Additionally, the concentration of psilocybin, psilocin and other tryptamines was enhanced when using vortex agitation during the extraction process. The research emphasizes that fresh fruiting bodies yield higher amounts of psilocin, while dried fruiting bodies retain more psilocybin, underlining the importance of processing and storage conditions for maintaining tryptamine stability and concentration. Additional information regarding the extraction of *P. cubensis* samples can be found in Goff et al. [12], where optimized extraction methods and analytical techniques were employed to maximize the recovery and quantification of psilocybin and psilocin from various strains of *P. cubensis*.

Several analytical methods have been employed to quantify psilocin and psilocybin in hallucinogenic mushrooms (Table 2). The most used technique is high-performance liquid chromatography (HPLC) coupled with various detectors, including ultraviolet (UV) [43,44,45,46], fluorescence [47], chemiluminescence [48] and mass spectrometry [12,49]. Additional techniques for psilocin and psilocybin analysis include gas chromatography–mass spectrometry (GC-MS) [50] and capillary electrophoresis [51]. The primary aim of these methods is to improve detection limits and selectivity, as these compounds are often present in trace amounts.

The detection of psilocybin and psilocin requires careful selection of an appropriate analytical technique based on factors such as sensitivity, specificity and compatibility with the sample matrix. Due to their superior sensitivity and specificity, GC-MS and HPLC-MS/MS are often preferred. However, GC-MS requires derivatization, which adds complexity to sample preparation. Furthermore, GC-MS is not recommended for psilocybin analysis because the compound undergoes rapid dephosphorylation to psilocin at high temperatures [47]. While HPLC-UV offers good resolution, it generally has lower sensitivity compared to MS, particularly for detecting low-concentration samples. Some HPLC-UV methods have achieved low limits of detection (LOD) and limits of quantitation (LOQ) through sample concentration techniques, such as evaporating the extraction solution and redissolving analytes in small volumes. However, HPLC-UV is more prone to matrix effects, making it less suitable for high-precision analyses. In addition, HPLC-fluorimetry and HPLC-chemiluminescence introduce additional complexity, as they require derivatization or post-column reactions. Capillary electrophoresis, while simpler, has lower sensitivity compared to chromatography-based methods.

In summary, the choice of analytical method depends on the required sensitivity and the nature of the sample. For high-precision quantification of psilocybin and its analogs, LC-MS/MS is the ideal choice. This method also has significant applications in clinical research and therapeutic contexts, particularly for the standardization of dosages. It enables detailed pharmacokinetic profiling of psilocybin, providing critical insights into its metabolic pathways and interactions in the human body. This level of precision is essential for ensuring consistent outcomes in clinical trials and optimizing therapeutic dosages for patient safety and efficacy [30,31].

## 6. Therapeutic Potential of Psilocybin and Mechanism of Action

Numerous studies have highlighted the therapeutic potential of psychedelics in clinical applications, particularly in treating mental health disorders. The most robust evidence is currently available for psilocybin, with major clinical trials showing its efficacy in treating major depressive disorder (MDD) and treatment-resistant depression (TRD), end-of-life distress and anxiety and addiction. As early as 2018 and 2019 [52,53], psilocybin was labeled as a breakthrough therapy by the US Food and Drug Administration (FDA) in the treatment of TRD and MDD following clinical trials of Compass Pathways and Usona, and extended access to patients was allowed [52,53]. Recently, in 2024, a psilocybin analogue developed by Cybin was also labeled a breakthrough therapy for MDD [54]. Currently, over 160 registered clinical trials with psilocybin also focus on several other disorders, e.g., obsessive-compulsive disorder (OCD), eating disorders, cluster headache, generalized anxiety, post-traumatic stress disorder (PTSD), chronic pain, etc. [55,56]. While the first recent trials were mainly conducted with a single dose and addressed safety and efficacy, a unified design across trials is currently lacking. Some studies use full psychedelic doses, others use dose escalation schemes, while yet others also work with microdosing (doses ca. 5–10 times lower than the full psychedelic dose) [57,58,59,60]. Most of the current ongoing patient trials have already implemented open-label extensions, so all patients can finally benefit from the drug studied. This enables answers to be found for questions on the safety of repeated administration and the duration of efficacy following multiple doses.

Nonetheless, several issues related to clinical trials with psychedelic drugs are currently being investigated both by scientists, industry and regulators [61]. The first issue is how to distinguish between the effect of the drug alone and the effect of the drug in combination with psychological support or psychotherapy. While psychological support is a framework to support the safety of patients, psychotherapy is an intervention. As regulators are typically expecting the effects to be clearly related to the drug, psychotherapy becomes a confounder. The second issue is unblinding, which primarily refers to the fact that due to the nature of their effects, psychoactive drugs typically unmask themselves with respect to both the patient and the accompanying person/therapist [62,63]. This, in turn, interacts with the level of expectations and generates additional bias. Other unanswered questions are whether the speed of onset, duration and intensity of psychotomimetic effects are relevant, whether these are desirable or negative side effects and what the role is of set and setting, preparedness of subjects, previous experience with psychedelic drugs, etc. Last but not least, little information exists on concomitant medications—whether psychedelics can be used as add-ons to standard medications or whether and which medications must be tapered off prior to the treatment [64,65]. These issues are difficult to address all at once, thus, more clinical trials are needed to address them separately. In light of the above, study designs of newer trials have been duly adapted: some use factorial designs [66] of clinical trials to control for expectations and unblinding, while others use active comparators or compare high versus low doses instead of using an inactive placebo, etc. [67,68]. Finally, most studies nowadays use open-label extension in their designs [56].

From the neurobiological point of view, there are four main mechanisms that most likely play a role in the therapeutic effect of psychedelics. The first is the direct effect on receptors, mainly 5-HT2A, which are rapidly downregulated following exposure to psychedelics. Other serotonergic receptors, such as 5-HT1A, 5-HT2C and 5-HT7, most likely also play a role [8,69,70,71,72]. The second and currently most important mechanism is the enhancement of neuroplasticity at the synaptic level, which is triggered by most psychedelics [73,74,75]. The third mechanism is related to the phenomenology of experience and the psychological impact of the experience induced by psychedelic drugs. Most studies consistently show that the intensity of experience, its positive valence or the ability to achieve mystical experiences are predictors of a long-lasting response [62,63]. In contrast, some companies are trying to develop non-psychedelic psychedelics that lack the psychotomimetic properties. To some extent this might work, e.g., with the prolonged release of ketamine formulations or esketamine in a nasal spray [76,77]. The last mechanism is believed to be linked to the rapid change in functional brain state based on analyses that evaluate the measures of complexity and/or connectivity of the brain and its networks in relation to psychological and cognitive flexibility. These hypotheses in general propose that psychedelics can switch the brain from a low-entropy state with rigid low cognitive/psychological flexibility towards a state with higher entropy and enhanced cognitive/psychological flexibility [72,78,79,80,81,82,83,84,85,86]. Overall, it seems that all these mechanisms are combined and have a synergistic role.

There is an ongoing discussion in the literature regarding the use of synthetic psilocybin over whole mushrooms or mycological extracts, as there could be potential synergistic effects of the substances contained in the whole mushroom or extracts [87]. Unfortunately, it is difficult to answer this question based on existing studies, as very few have used whole mushrooms or extracts and even fewer have compared either of these to pure psilocybin. In humans, only one study to date has compared psilocybin with whole *P. cubensis* and a mycological extract in a patient population that received psilocybin-assisted therapy for end-of-life distress. Patients reported that the effects of the three preparations were very similar in terms of acute effects and therapeutic efficacy, with the authors concluding that ‘synthetic psilocybin was said to feel less natural compared to organic forms and the overall quality of experience of synthetic psilocybin was inferior to the organic forms’ [88]. In a double-blind study with microdosing of dried, encapsulated *P. cubensis* mushrooms, authors reported that some subjects were able to recognize the acute effects of the drug; however, the authors concluded that they found no enhancing impact on well-being, creativity or cognitive function [57]. One study in mice, evaluating the potential efficacy against OCD (marble-burying behavior as an OCD-like symptom), showed that an extract from *P. argentipes* was more effective in reducing the marble-burying compared to pure psilocybin and that this effect was comparable to fluvoxamine, a drug typically used to treat OCD. The effect of the extract appeared at a calculated dose of 0.24 mg/kg psilocybin compared to 1.5 mg/kg of pure psilocybin [89]. Another study compared whole psilocybin mushroom extract (PME, without specifying the strain) with psilocybin on the head twitch response (HTR, a marker of 5-HT2A activity), neuroplasticity-related synaptic proteins and frontal cortex metabolomic profiles in C57Bl/6j mice. They found comparable effects on HTR, but a Western blot showed a more pronounced and prolonged effect of the whole extract on the expression of proteins in all three structures evaluated (frontal cortex, striatum, hippocampus) [34]. Other preclinical studies have also shown more pronounced effects of psilocybin extracts than psilocin/psilocybin alone, thus supporting the potential of synergistic effects of all components of *Psilocybe* mushrooms over pure psilocybin [90,91,92,93,94].

## 7. Regulatory Considerations

Since 1971, the United Nations (UN) Convention on Psychotropic Substances has designated psilocybin and most other psychedelics as Schedule I drugs: substances or chemicals that have no currently accepted medical use and have a high potential for abuse. This greatly complicates matters when seeking to market them as therapeutics, which by definition of this class is not possible. Based on a recent internet search that focused on https://psychedelicalpha.com [95], few locations around the globe currently have legislation that looks favorably on the use of *Psilocybe* mushrooms and psilocybin. One of the world’s largest countries, where psilocybin mushrooms are completely legal, is Brazil; however, psilocin and psilocybin are scheduled. Similarly, in the Caribbean, Jamaica and Saint Vincent and the Grenadines also have regulations enabling the use of psilocybin and other psychedelics for therapeutic purposes. The Netherlands has a long history of the legal sale of psilocybin-containing truffles (mycelia), although psilocybin itself has not been legalized. Also, Austria, Nepal and the British Virgin Islands do not regulate psilocybin mushrooms. In some countries, e.g., the Czech Republic, Spain and Portugal and several countries in Latin America, the use and possession of limited amounts of scheduled drugs, including psilocybin and mushrooms, is legal; however, any trafficking or sale is illegal. According to the International Center for Ethnobotanical Education, Research and Service (ICEERS), Spain has a tendency not to schedule naturally occurring drugs [96]. Preparations (e.g., decoctions for oral use) made from plants containing those active ingredients are also not under international control’, although psilocybin and psilocin are scheduled drugs. Additionally, in Spain, ceremonies with natural compounds such as ayahuasca, San Pedro and mushrooms seem to be in a gray zone and are widely offered over the internet. This is possible because Spanish regulations state that the use of drugs on private property is not a criminal offense [97,98]. Other countries, e.g., Switzerland and the US, permit the use of psilocybin mushrooms for ceremonial religious purposes. In the rest of the world, psilocybin, psilocin and mushrooms are mostly designated as scheduled drugs, in line with the 1971 UN Convention on Psychotropic Substances.

However, today there are a number of countries that have overcome or solved this issue for therapeutic purposes. The country with the longest continuous history of therapeutic use of psychedelics is Switzerland, where a legal framework has been created for using psychedelics in the context of psychedelic-assisted therapy by licensed psychotherapists. Since 1985, these therapists are associated under the Swiss Medical Society for Psycholytic Therapy (Schweizerische Ärztegesellschaft für psycholytische Therapie, SAPT) and with over 60 members, over a thousand therapeutic psychedelic/psycholytic sessions have been conducted to date [99]. Probably one of the first regulatory changes took place in the US, where several states have made psychedelics such as psilocybin and MDMA or mushrooms legal for therapeutic use, e.g., Oregon, California and Colorado. Moreover, approximately 18 US cities have decriminalized psychedelics [100,101,102]. Since 2020, Canada has permitted the use of psilocybin and MDMA for medicinal purposes in personal exemptions and since 2022 in a special access program. The special access program also allows production of these drugs for medicinal purposes [103,104]. Since 2019, Israel has also allowed compassionate use of psychedelics [105], while in 2023, Australia approved a new regulation that to permits the use of psilocybin and MDMA for the treatment of TRD and PTSD under strict conditions [106].

The European Medicines Agency (EMA) and the U.S. National Institutes of Health (NIH) share a similar perspective on psilocybin as a therapeutic intervention, recognizing its potential but underscoring the need for more rigorous research. The EMA acknowledges the resurgence of interest in psychedelics for treating conditions such as treatment-resistant depression and PTSD but emphasizes that challenges such as dosing, placebo controls and safety concerns, such as anxiety and psychological distress, must be addressed prior to widespread use. Regulatory approval requires robust clinical evidence on both efficacy and safety [107]. Similarly, Xi et al. [108], in a comprehensive evaluation from the first-ever NIH congress on psychedelics, highlight the therapeutic promise of psilocybin in psychiatric conditions but stress the urgent need for evidence-based guidelines for healthcare professionals, citing research gaps and regulatory challenges. Both agencies are thus aligned in their cautious optimism, advocating for more rigorous research and clearer regulatory frameworks before psilocybin can be integrated into clinical practice.

## 8. Future Directions

Future research on *P. cubensis* must also address the significant strain variability in psychoactive compound concentrations, particularly psilocybin and psilocin. Different strains, such as ‘Creeper’, ‘Blue Meanie’ and ‘Thai Cubensis’, have been shown to produce varying levels of these compounds, which can affect their efficacy in clinical settings. This highlights the importance of strain selection and the development of standardized cultivation methods to ensure consistency across batches. Establishing reliable quality control measures in the cultivation of *P. cubensis* strains will be crucial for maintaining consistent potency and safety, a vital step for their integration into pharmaceutical treatments.

In addition, future research should explore the potential of cultivating *P. cubensis* mycelium rather than relying on fruiting bodies for psilocybin extraction. Mycelium-based cultivation presents a promising alternative, as it can offer more controlled, repeatable results. Mycelium tends to grow more uniformly than fruiting bodies, which may lead to more consistent concentrations of psychoactive compounds. Furthermore, mycelium cultivation could be more conducive to regulatory approval, such as from the FDA, given its ease of standardization and potential for large-scale production. This shift could be a critical advancement for the pharmaceutical industry, offering a more reliable, scalable method for producing psilocybin-based therapies. However, the structures of psilocybin and related alkaloids are not complex for chemical synthesis and several methods for their synthesis have been published and patented. It is important to note that pure compounds, whether obtained via chemical synthesis or biotechnological processes, are more likely to align with regulatory requirements from agencies such as the FDA due to their precise characterization and consistent composition. While fungal cultivation introduces variability, it also offers potential advantages such as exploring secondary metabolites and synergistic effects, which could have unique therapeutic implications.

The challenge moving forward will be to optimize mycelial cultivation to match or surpass the psychoactive compound yield of fruiting bodies while maintaining bioavailability and therapeutic efficacy. Researchers will need to refine extraction methods and develop robust analytical techniques to ensure the potency and purity of mycelium-based products. Furthermore, understanding how different compound compositions and concentrations affect the brain, including potential synergistic interactions (the ‘entourage effect’), remains a critical area of research. Such insights will not only improve therapeutic outcomes by tailoring treatments to specific conditions but also help mitigate adverse effects, such as anxiety or ‘bad trips’, while promoting more positive therapeutic experiences. Addressing these challenges will be key to achieving regulatory approval and making psilocybin treatments widely available, opening up new possibilities for treating psychiatric disorders with precision and consistency.

Another promising direction for research is understanding the tetrapolar mating system in *Psilocybe* species, including *P. cubensis*. This system, which involves compatibility at two unlinked genetic loci, reduces inbreeding and helps maintain genetic diversity. As shown by McTaggart et al. [109], studying mating systems and recombination in *Psilocybe subaeruginosa* can inform selective breeding strategies to enhance traits like psilocybin production. Additionally, their discovery of gene duplications in the psilocybin biosynthesis pathway highlights the potential for variability in psilocybin production, which could be used to develop optimized strains. Applying these findings could benefit both fruiting body cultivation and mycelium-based production in submerged fermentation systems, making these approaches more efficient and scalable for pharmaceutical use.

While significant advances have been made in understanding the genetic basis of psilocybin biosynthesis, including the identification of the psilocybin biosynthetic gene cluster, critical knowledge gaps remain. One of the most pressing issues for future investigation is the role of regulatory sequences controlling the expression of key genes involved in psilocybin production. Regulatory elements, such as promoters, enhancers and repressors, likely play a pivotal role in modulating the activity of these biosynthetic pathways in response to environmental and developmental cues. Identifying and characterizing these regulatory sequences could provide deeper insights into how *Psilocybe* species control the production of psychoactive compounds, opening up new avenues for genetic manipulation.

By understanding how gene expression is fine-tuned, researchers will be able to optimize psilocybin yields through targeted genetic engineering or selective breeding. Furthermore, investigating regulatory sequences across different strains and species could shed light on the genetic and environmental factors that contribute to the observed variability in psilocybin and psilocin concentrations. This knowledge could be instrumental in developing strains with enhanced or tailored biosynthetic capacities, paving the way for scalable, consistent, cost-effective production methods for pharmaceutical applications. As research continues to bridge the gap between genetic mechanisms and applied cultivation practices, the integration of regulatory sequence analysis will be essential to unlocking the full therapeutic potential of psilocybin-producing fungi.

## 9. Conclusions

From an applied perspective, the expanding body of research on *Psilocybe* species in general and *P. cubensis* strains in particular, alongside their therapeutic applications, is unlocking new pathways for treating psychiatric conditions. Future research should focus on refining cultivation practices, enhancing analytical methods and exploring the genetic basis of psilocybin biosynthesis, including understanding the gene regulation mechanisms that influence the production of psychoactive compounds across different strains. Our findings indicate growing scientific interest, with publication rates on psilocybin genetics significantly increasing from single annual studies in the early 2000s to over 25 publications in 2023 alone. This trend underscores the expanding research momentum on the genetic underpinnings and therapeutic potential of psilocybin-producing fungi. Comprehensive strain selection and genetic improvements could further optimize therapeutic efficacy and safety.

Additionally, the diversity in cultivation techniques, such as the promising approach of submerged fermentation for mycelium production, offers a controlled, scalable alternative to traditional fruiting-body cultivation. Submerged fermentation enables precise environmental control and consistency in producing psychoactive compounds, making it suitable for pharmaceutical applications where standardization is essential. Recent studies have shown that although mycelium typically contains lower concentrations of psilocybin than fruiting bodies, it also produces unique secondary metabolites, indicating its distinct biosynthetic potential. Optimizing the cultivation of both mycelium and fruiting bodies is crucial to maximizing the yield and therapeutic efficacy of psilocybin-based treatments.

Strain variability and biochemical diversity also play a significant role in the therapeutic applications of psilocybin. Studies indicate considerable inter- and intra-strain variability in the concentrations of psychoactive compounds, which is influenced by genetic and environmental factors. Recent findings suggest that advances in genetic mapping and the identification of the psilocybin biosynthetic gene cluster are laying the groundwork for targeted strain development. The ability to modify specific genetic pathways could enable the cultivation of strains with defined psychoactive profiles tailored for therapeutic applications.

Future directions should emphasize the integration of cultivation practices, genetic screening and analytical techniques to achieve consistent, reproducible psilocybin production. Establishing standardized protocols for strain cultivation, coupled with precise quantification methods using advanced chromatography and mass spectrometry techniques, will be essential for advancing therapeutic applications and ensuring patient safety. By refining these processes and building on the momentum of recent research, we can turn psilocybin into a reliable, well-regulated, innovative tool for psychiatric care.

## Figures and Tables

**Table 1:** Quantification of psilocybin and psilocin in *Psilocybe cubensis* strains.

#	*P. cubensis* Strain Name	Fruiting Body or Mycelium	Origin of Specimen (Wild or Lab-Grown, Location)	Growth Conditions	Psilocybin (mg/g)	Psilocin (mg/g)	Ref.
1	Thai Cubensis	Fruiting body	Scottsdale Research Institute (lab grown)	NA	8.1 ± 0.7	0.7 ± 0.2	[12]
2	Blue Meanie	Fruiting body	Scottsdale Research Institute (lab grown)	NA	11.8 ± 0.4	0.36 ± 0.15	[12]
3	Creeper	Fruiting body	Scottsdale Research Institute (lab grown)	NA	13 ± 3	0.24 ± 0.07	[12]
4	B+	Fruiting body	Scottsdale Research Institute (lab grown)	NA	11.1 ± 2.5	0.22 ± 0.09	[12]
5	Texas Yellow	Fruiting body	Scottsdale Research Institute (lab grown)	NA	10.8 ± 0.8	0.24 ± 0.05	[12]
6	*P. cubensis*	Fruiting body	Lab-grown	Controlled conditions, medium: mixture of cow dung and rice grains with water	0.45–6.3	1.1–2.5	[16]
7	M.R.	Fruiting body	A spore print taken from the Amazon region near Pucalpa, Peru	The cultures were grown on agar and transferred to rye medium in jars	4.2–9.7	0–0.35	[17]
8	Equadorian strain	Fruiting body	A spore print taken from the Amazon region near Pucalpa, Peru	The cultures were grown on agar and transferred to rye medium in jars	4.7–7.6	0–0.4	[17]
9	Amazon strain	Fruiting body	A spore print taken from the Amazon region near Pucalpa, Peru	The cultures were grown on agar and transferred to rye medium in jars	5.7	0–0.1	[17]
10	*P. cubensis* voucher specimens WTU-F-054871	Fruiting body	University of Washington Herbarium (1970)	NA	10.5–15.44	1.9–2.11	[10]
11	*P. cubensis* voucher specimens WTU-F-011258	Fruiting body	University of Washington Herbarium (1974)	NA	16.33–17.3	0.4–0.5	[10]
12	*P. cubensis* voucher specimens WTU-F-054869	Fruiting body	University of Washington Herbarium (1978)	NA	18.3–19.9	0.44–1.5	[10]
13	*P. cubensis* var. Ecuadorian	Fruiting body	Lab grown from an online supplier (Spaceseed S.L., El Escorial, Spain)	Controlled conditions, humidity above 85%. Temperature 20–25 °C	0.490 ± 0.047 (Total Indole Alkaloids)	NA	[18]
14	*P. cubensis* var. B+	Fruiting body	Lab grown from an online supplier (Spaceseed S.L., El Escorial, Spain)	Controlled conditions, humidity above 85%. Temperature 20–25 °C	1.609 ± 0.081 (Total Indole Alkaloids)	NA	[18]
15	*P. cubensis* (Earle) Singer	mycelium	NRRL A-9109 (National Center for Agricultural Utilization Research)	Submerged culture on a glucose-based medium (1%) under controlled laboratory conditions.	0.1–1.1	NA	[19]
16	*P. cubensis* (Earle) Singer.	mycelium	The Northern Regional Research Laboratory (NRRL) Peoria, Illinois, the NRRL number of the culture A-9109	Submerged culture, 25 °C, medium containing glucose, yeast extract, salts, pH 4.5	0.3–5.2	NA	[20]
17	*P. cubensis*	Fruiting body	Wild	Natural growth on buffalo/cattle excrement	1–3.6	2–6	[21]
18	*P. cubensis* (Mexican strain)	Fruiting body	Wild	NA	1.2–1.5	0.5–5	[21]
19	*P. cubensis* (Amazonian strain)	Fruiting body	Wild	NA	1.2–1.5	1.0–3.3	[21]
20	*P. Cubensis* #1	Fruiting body	Imported	Imported dried sample	11	2.2	[22]
21	*P. Cubensis* #2	Fruiting body	Local kit	Cultivation kit (Japan)	7.5	2.5	[22]
22	*P. Cubensis* #3	Fruiting body	Wild (Japan)	Collected locally	6.9	4.2	[22]
23	*P. Cubensis* #4	Fruiting body	Imported	Imported dried sample	3.7	1.8	[22]
24	*P. Cubensis* #5	Fruiting body	Local kit	Cultivation kit (Japan)	4.7	1.5	[22]
25	*P. Cubensis* #6	Fruiting body	Wild (Japan)	Collected locally	13	1.4	[22]
26	*P. Cubensis*	Fruiting body	lab grown	Cow dung/rice grain mixture (cultivated)	6.3	1.1	[23]

NA represents not available data.

**Table 2:** Methods for quantitative analysis of psilocin and psilocybin in fungal material.

Analyte	Detection Technique	LOD, LOQ	Method Conditions	Ref.
Psilocin, psilocybin	HPLC-UV	Psilocybin LOD: 0.07 ng/mg; psilocin LOD: 0.13 ng/mg	LiChrosorb RP-18 (250 mm, i.d. 5 μm), semipreparative column: LiChrosorb RP-18 (500 mm, i.d. 10 μm). Eluents: isocratic with ethanol-UPW-acetic acid (20:79.2:0.8). Wavelength: 267 nm. Extraction solutions evaporated to dryness and re-dissolved in methanol.	[46]
Psilocin, psilocybin	HPLC-UV	Psilocybin LOD: 0.5 ng/mg; psilocin LOD: 0.4 ng/mg	Spherisorb ODS-1 (250 mm × 4.6 mm, i.d. 5 μm) column. Eluents: UPW + 0.3 M ammonium acetate (pH 8), methanol + 0.3 M ammonium acetate. Wavelength: 269 nm. Extract concentrated under a stream of N_2_.	[43]
Psilocin, psilocybin	HPLC-UV (cation exchange)	Psilocybin LOD: 70 ng/mg, LOQ: 280 ng/mg; psilocin LOD: 60 ng/mg, LOQ: 220 ng/mg	Luna SCX 100A (150 mm × 4.60 mm, i.d. 5 μm) cation exchange column. Eluents: 95:5 mixture of buffer (50 mM KH_2_PO_4_, 100 mM NaCl) and ethanol (pH 3.0 with 50 mM H3PO_4_). Wavelength: 220 nm.	[45]
Psilocin, psilocybin	HPLC-UV	Psilocybin LOD: 10 ng/mL, LOQ: 100 ng/mL; psilocin LOD: 50 ng/mL, LOQ: 100 ng/mL	Supelcosil TM LC-SCX, (25 cm × 3.0 mm, i.d. 5 μm) column. Eluents: NaCl 100 mM + KH_2_PO_4_ 50 mM (pH 3 with 85% H_3_PO_4_) and methanol. Wavelength: 220 nm.	[44]
psilocybin	HPLC-fluorometry	LOQ: 4.4 ng/mg	Mightysil RP-18 GP column (150 mm × 4.6 mm, i.d. 3 μm). Eluent: 50 mM ammonium acetate and acetonitrile. Wavelength: 539 nm (excitation at 321 nm). Extraction solutions evaporated to dryness and re-dissolved in methanol. Derivatization method: DNS-ED at 60 °C.	[47]
Psilocin, psilocybin	HPLC-chemiluminesce	Psilocyn LOD: 2.4 ng/mL, psilocybyn LOD: 0.9 ng/mL	Synergi Max-RP C12 (150 mm × 4.6 mm, i.d. 4 μm) column. Post-column reagents: potassium permanganate and tris (2,20-bipyridyl) ruthenium (II) merged before photomultiplier tube detection.	[48]
Psilocin, psilocybin	LC-MS/MS	LOD: 3 ng/mg, LOQ: 10 ng/mg for both psilocin and psilocybin	Poroshel 120 SB-C18 column (100 mm × 2.1 mm, i.d. 2.7 μm). Eluents: UPW + 0.1% formic acid and acetonitrile. Extraction solution concentrated by evaporation to dryness and re-dissolved prior to analysis.	[49]
Psilocin, psilocybin	LC-MS/MS	Psilocibyn: LOD: 0.3 ng/mg (1.5 ng/mL), LOQ: 1 ng/mg (5 ng/mL). Psilocin: LOD: 0.03 ng/mg (0.15 ng/mL), LOQ: 0.1 ng/mg (0.5 ng/mL)	Biphenyl column (50 mm × 2.1 mm, i.d. 2.7 μm). Eluents: UPW + 0.1% formic acid and acetonitrile + 0.1% formic acid	[12]
Psilocin, psilocybin	GC-MS	Psilocybin LOD: 100 ng/mg; psilocyn LOD: 10 ng/mg	HP-5 MS fused-silica capillary column (30 m, i.d. 0.25 mm, 0.25 μm film thickness), helium as the carrier gas. Derivatization method with MSTFA at 70 °C.	[50]
Psilocybin	capillary zone electrophoresis	LOD: 45 ng/mg (0.0009 mg/mL), LOD: 225 ng/mg (0.0045 mg/mL)	Column: 57 cm × 50 µm fused-silica capillary for electrophoresis with hydrodynamic or electrokinetic injection. Operation: 25 kV, 25 °C, 220 nm and 10 mM borate–phosphate (pH 11.5) buffer.	[51]

MSTFA: chloroform and N-Methyl-N-(trimethylsilyl)-2,2,2-trifluoroacetamide, DNS-ED: 5-dimethylaminonaphthalene-1-[N-(2-aminoethyl)] sulfonamide, LOD: limit of detection, LOQ: limit of quantitation, UPW: ultrapure water, HPLC(-UV): high performance liquid chromatography (with ultraviolet detection), LC-MS/MS: liquid chromatography with tandem mass spectrometry, GC-MS: gas chromatography with mass spectroscopy.
