# Correction: Kurzbaum et al. Exploring *Psilocybe cubensis* Strains: Cultivation Techniques, Psychoactive Compounds, Genetics and Research Gaps. *J. Fungi* 2025, *11*, 99

**DOI:** 10.3390/jof12040265

**Published:** 2026-04-07

**Authors:** Eyal Kurzbaum, Tomáš Páleníček, Amiel Sharchaton, Sara Azerrad, Yaron Dekel

**Affiliations:** 1Water Science Department, Tel-Hai College, Upper Galilee 1220800, Israel; 2Shamir Research Institute, University of Haifa, P.O. Box 97, Qatzrin 1290000, Israel; eyal2@gri.org.il (A.S.); saraa@gri.org.il (S.A.); yarond@gri.org.il (Y.D.); 3Department of Geography and Environmental Studies, University of Haifa, Mount Carmel, Haifa 3498838, Israel; 4National Institute of Mental Health, 250 67 Klecany, Czech Republic; tomas.palenicek@nudz.cz; 53rd Faculty of Medicine, Charles University, 100 00 Prague, Czech Republic; 6The Natural Resources and Environmental Research Center-NRERC, University of Haifa, Mount Carmel, Haifa 3498838, Israel; 7The Cheryl Spencer Department of Nursing, The Cheryl Spencer Institute of Nursing Research, University of Haifa, Haifa 3498838, Israel; 8The Cheryl Spencer Institute of Nursing Research, University of Haifa, Haifa 3498838, Israel

## Error in Table

In the original publication [1], there was a mistake in Table 1 as published. Several errors occurred during the compilation and management of the data. Some errors were only typing errors and others putting “±” instead of just “−” and putting the bigger number on the right and the smaller on the left. We have now carefully rechecked the table and believe that all inaccuracies have been corrected. The corrected Table 1 appears below. The authors state that the scientific conclusions are unaffected. This correction was approved by the Academic Editor. The original publication has also been updated.

In addition, a correction is needed in the author name Amiel Shrchaton: Amiel Sharchaton—‘a’ should be after Sh.

## References

With this correction, the order of some references has been adjusted accordingly. The authors state that the scientific conclusions are unaffected. This correction was approved by the Academic Editor. The original publication has also been updated.

## Figures and Tables

**Table 1 jof-12-00265-t001:** Quantification of psilocybin and psilocin in *Psilocybe cubensis* strains.

#	*P. cubensis* Strain Name	Fruiting Body or Mycelium	Origin of Specimen (Wild or Lab-Grown, Location)	Growth Conditions	Psilocybin (mg/g)	Psilocin (mg/g)	Ref.
1	Thai Cubensis	Fruiting body	Scottsdale Research Institute (lab grown)	NA	8.1 ± 0.7	0.7 ± 0.2	[12]
2	Blue Meanie	Fruiting body	Scottsdale Research Institute (lab grown)	NA	11.8 ± 0.4	0.36 ± 0.15	[12]
3	Creeper	Fruiting body	Scottsdale Research Institute (lab grown)	NA	13 ± 3	0.24 ± 0.07	[12]
4	B+	Fruiting body	Scottsdale Research Institute (lab grown)	NA	11.1 ± 2.5	0.22 ± 0.09	[12]
5	Texas Yellow	Fruiting body	Scottsdale Research Institute (lab grown)	NA	10.8 ± 0.8	0.24 ± 0.05	[12]
6	*P. cubensis*	Fruiting body	Lab-grown	Controlled conditions, medium: mixture of cow dung and rice grains with water	0.45–6.3	1.1–2.5	[16]
7	M.R.	Fruiting body	A spore print taken from the Amazon region near Pucalpa, Peru	The cultures were grown on agar and transferred to rye medium in jars	4.2–9.7	0–0.35	[17]
8	Equadorian strain	Fruiting body	A spore print taken from the Amazon region near Pucalpa, Peru	The cultures were grown on agar and transferred to rye medium in jars	4.7–7.6	0–0.4	[17]
9	Amazon strain	Fruiting body	A spore print taken from the Amazon region near Pucalpa, Peru	The cultures were grown on agar and transferred to rye medium in jars	5.7	0–0.1	[17]
10	*P. cubensis* voucher specimens WTU-F-054871	Fruiting body	University of Washington Herbarium (1970)	NA	10.5–15.44	1.9–2.11	[10]
11	*P. cubensis* voucher specimens WTU-F-011258	Fruiting body	University of Washington Herbarium (1974)	NA	16.33–17.3	0.4–0.5	[10]
12	*P. cubensis* voucher specimens WTU-F-054869	Fruiting body	University of Washington Herbarium (1978)	NA	18.3–19.9	0.44–1.5	[10]
13	*P. cubensis* var. Ecuadorian	Fruiting body	Lab grown from an online supplier (Spaceseed S.L., El Escorial, Spain)	Controlled conditions, humidity above 85%. Temperature 20–25 °C	0.490 ± 0.047 (Total Indole Alkaloids)	NA	[18]
14	*P. cubensis* var. B+	Fruiting body	Lab grown from an online supplier (Spaceseed S.L., El Escorial, Spain)	Controlled conditions, humidity above 85%. Temperature 20–25 °C	1.609 ± 0.081 (Total Indole Alkaloids)	NA	[18]
15	*P. cubensis* (Earle) Singer	mycelium	NRRL A-9109 (National Center for Agricultural Utilization Research)	Submerged culture on a glucose-based medium (1%) under controlled laboratory conditions.	0.1–1.1	NA	[19]
16	*P. cubensis* (Earle) Singer.	mycelium	The Northern Regional Research Laboratory (NRRL) Peoria, Illinois, the NRRL number of the culture A-9109	Submerged culture, 25 °C, medium containing glucose, yeast extract, salts, pH 4.5	0.3–5.2	NA	[20]
17	*P. cubensis*	Fruiting body	Wild	Natural growth on buffalo/cattle excrement	1–3.6	2–6	[21]
18	*P. cubensis* (Mexican strain)	Fruiting body	Wild	NA	1.2–1.5	0.5–5	[21]
19	*P. cubensis* (Amazonian strain)	Fruiting body	Wild	NA	1.2–1.5	1.0–3.3	[21]
20	*P. Cubensis* #1	Fruiting body	Imported	Imported dried sample	11	2.2	[22]
21	*P. Cubensis* #2	Fruiting body	Local kit	Cultivation kit (Japan)	7.5	2.5	[22]
22	*P. Cubensis* #3	Fruiting body	Wild (Japan)	Collected locally	6.9	4.2	[22]
23	*P. Cubensis* #4	Fruiting body	Imported	Imported dried sample	3.7	1.8	[22]
24	*P. Cubensis* #5	Fruiting body	Local kit	Cultivation kit (Japan)	4.7	1.5	[22]
25	*P. Cubensis* #6	Fruiting body	Wild (Japan)	Collected locally	13	1.4	[22]
26	*P. Cubensis*	Fruiting body	lab grown	Cow dung/rice grain mixture (cultivated)	6.3	1.1	[23]

NA represents not available data.
